# Guillain-Barré syndrome following *Escherichia coli* meningitis after cupping therapy: a case report

**DOI:** 10.3389/fimmu.2026.1839976

**Published:** 2026-05-22

**Authors:** Anping Liu, Yuling Liu, Lili Shi, Binyuan Xiong, Dan Zhi, Haizhen Duan, Anyong Yu

**Affiliations:** 1 Department of Emergency Medicine, Affiliated Hospital of Zunyi Medical University, Zunyi, Guizhou, China; 2 Zunyi Medical University College of Science and Technology, , Zunyi, China; 3 Zunyi Medical University, Zunyi, China

**Keywords:** acupuncture, bacterial meningitis, case report, cupping therapy, *Escherichia coli*, Guillain-Barré syndrome, systemic lupus erythematosus

## Abstract

Guillain-Barré syndrome (GBS) is an acute immune-mediated polyneuropathy, whereas Escherichia coli meningitis following cupping and acupuncture is a rare but severe infection. GBS as a neurological complication after E. coli meningitis is exceedingly uncommon. We report the case of a 53-year-old woman with systemic lupus erythematosus and chronic renal failure who developed headache, vomiting, and altered consciousness four days after undergoing cupping and acupuncture for low back pain. Cerebrospinal fluid (CSF) analysis and next-generation sequencing confirmed E. coli meningitis. Although meningeal signs improved with targeted antibiotic therapy, she subsequently developed progressive flaccid quadriparesis, ophthalmoparesis, and areflexia. Further evaluation revealed CSF albuminocytologic dissociation, positive serum anti-GM1 and anti-GT1a IgG antibodies, and electromyographic findings consistent with acute demyelinating polyneuropathy, leading to a diagnosis of GBS. The patient’s neurological symptoms improved significantly following intravenous immunoglobulin therapy. This case illustrates that E. coli meningitis, potentially introduced via cupping or acupuncture, can trigger GBS as a severe neurological sequela. It also underscores the importance of considering GBS in the differential diagnosis when patients with bacterial meningitis develop progressive, unexplained flaccid paralysis during or after treatment. Early recognition, timely immunomodulation, and sustained rehabilitation are crucial for reducing disability.

## 1 Background

Guillain-Barré syndrome (GBS) is an acute inflammatory polyradiculoneuropathy typically triggered by a preceding infection. While pathogens such as Campylobacter jejuni, cytomegalovirus, and Zika virus are commonly implicated (1), other infectious agents, including Escherichia coli, have also been associated with GBS, primarily following urinary tract infections (2). However, its occurrence after pyogenic bacterial meningitis, particularly that caused by E. coli, is exceedingly rare. This report presents a confirmed case in which the patient developed Escherichia coli meningitis following cupping and acupuncture, and subsequently developed Guillain-Barré syndrome (GBS) during the convalescent phase, thereby expanding the known spectrum of triggers for GBS.

## 2 Case report

A 53-year-old woman presented to the emergency department with a 10-hour history of headache, vomiting, and progressive drowsiness. Her past medical history included hypertension, chronic renal failure, prior cerebral infarction, and systemic lupus erythematosus (SLE), which had been diagnosed three months earlier. Following the diagnosis of SLE, she was initially treated with prednisone 60 mg/day and hydroxychloroquine 200 mg/day. After four weeks, prednisone was tapered by 5 mg per week over seven weeks, reaching 25 mg/day by the time of this admission. She had not received any other immunosuppressive agents. Four days before admission, she had undergone cupping, acupuncture, and topical herbal application for low back pain. The patient’s family recalled that the acupuncture involved multiple insertions in the lumbar and paravertebral region, and cupping was applied to the same area, although details regarding the type of cups, needle materials, or disinfection protocols were not available.

### 2.1 Initial examination and investigations

**Figure 1:**
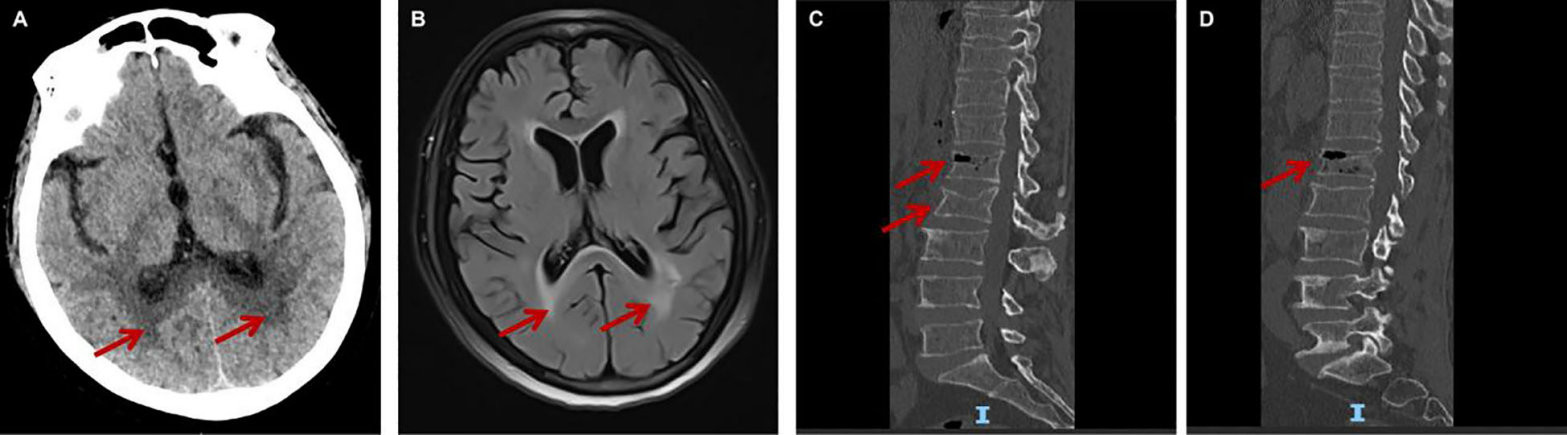
Imaging findings. **(A, B)** Non-contrast brain CT and MRI scans obtained after admission, showing interstitial cerebral edema and old lacunar infarcts. **(C, D)** Non-contrast lumbar spine CT obtained after admission, demonstrating bone marrow edema, gas accumulation or osseous destruction at the T12 and L1 vertebrae.

On admission, she was afebrile (36.3 °C), tachycardic (heart rate 98 bpm), tachypneic (respiratory rate 29 bpm), and normotensive (blood pressure 138/88 mmHg). Neurological examination revealed drowsiness. Her left pupil was small (approximately 2 mm in diameter) but reactive to light; the right pupil could not be assessed due to a prior ocular injury. Neck stiffness is present with a two-finger breadth limitation. Kernig’s sign and Brudzinski’s sign are positive. Muscle strength in all four limbs was graded as 5/5 with normal tone; sensation was intact; and pathological reflexes were absent. Initial laboratory findings were remarkable for leukocytosis (WBC 20.60×10^9^/L, neutrophils 95%), acute kidney injury (creatinine 394 μmol/L), hyperkalemia (K^+^ 6.01 mmol/L), elevated CRP (198 mg/L), and coagulopathy with elevated D-dimer (4 μg/mL). Cultures of the patient’s stool, urine, and sputum yielded no growth of organisms. Brain CT and MRI scans showed interstitial cerebral edema and old lacunar infarcts (**Figures 1A, B**). Lumbar spine CT showed bone marrow edema, gas accumulation or osseous destruction at the T12 and L1 vertebrae (**Figures 1C, D**). Lumbar puncture demonstrated a dramatically elevated CSF opening pressure (>400 mmH_2_O), with turbid, xanthochromic CSF showing pleocytosis (WBC 7480×10^6^/L), markedly elevated protein (7641 mg/L), and low glucose (0.2 mmol/L).

### 2.2 Initial diagnosis and management

Based on the clinical presentation and examination findings, the preliminary diagnoses were: severe piogenic meningitis, sepsis, and possible discitis. To secure the airway and prevent aspiration, endotracheal intubation and mechanical ventilation were immediately initiated. Prior to the availability of etiological results, empirical broad-spectrum antibiotic therapy with meropenem was commenced. Intravenous corticosteroids (methylprednisolone) were administered to control intracranial inflammation and reduce cerebral edema, concurrent with mannitol for dehydration and intracranial pressure reduction, supplemented by comprehensive life support and organ function maintenance. On hospital day 6, CSF next-generation sequencing results identified Escherichia coli-specific sequences (815 reads), confirming the causative pathogen. Following this comprehensive treatment regimen, the patient’s systemic inflammatory markers (e.g, CRP, PCT) and CSF white blood cell count showed a progressive decline, indicating initial control of the infection.

### 2.3 Clinical deterioration and diagnostic pivot

On hospital day 18, after initial stabilization and weaning from mechanical ventilation, she developed a new fever (38.5 °C) and increased production of yellow sputum. Concurrently, she manifested profound neurological findings inconsistent with her initial central nervous system infection: bilateral ptosis, flaccid quadriparesis (muscle strength 2/5), and diminished muscle tone. While multidrug-resistant Acinetobacter baumannii pneumonia was identified and treated with tigecycline, the flaccid paralysis raised suspicion for a secondary neuromuscular disorder. Given the dissociation between improving CSF pleocytosis and persistently elevated protein levels (albuminocytological dissociation), GBS was strongly suspected.

### 2.4 Confirmation of GBS and specific treatment

**Figure 2:**
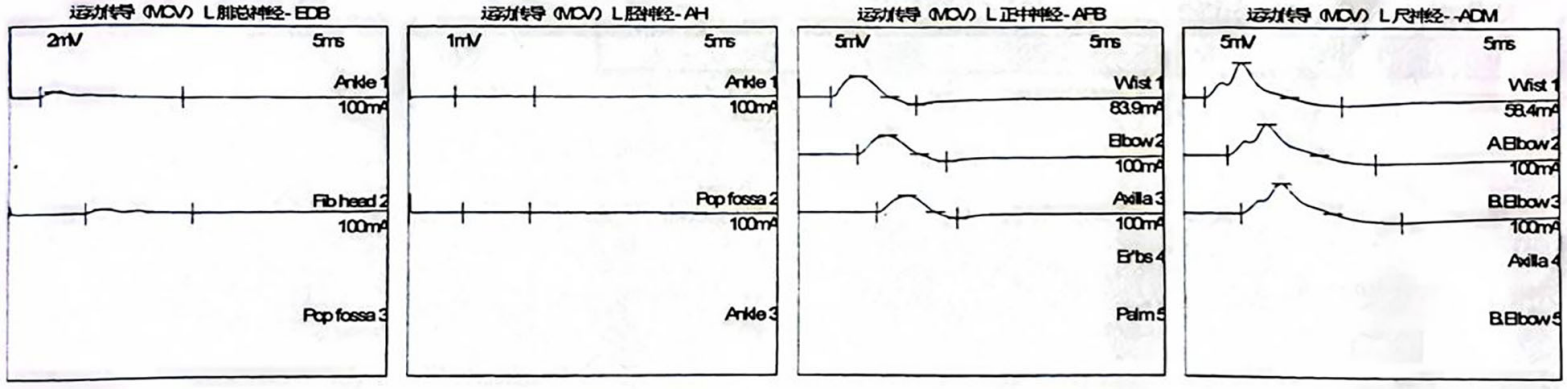
Motor nerve conduction study.

**Figure 3:**
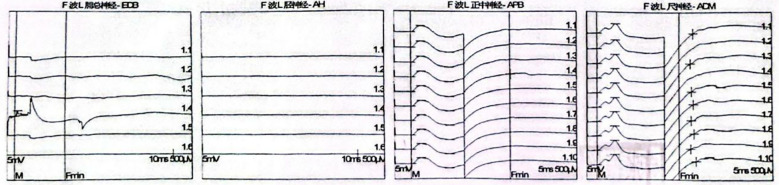
F-wave.

Electromyography and nerve conduction studies revealed a generalized peripheral polyneuropathy affecting all four limbs, characterized by demyelinating changes predominantly involving motor fibers (**Figures 2**, **3**, **Table 1**). Serological testing was positive for anti-GM1 and anti-GT1a IgG antibodies (**Table 2**). A repeat lumbar puncture confirmed albuminocytologic dissociation (white blood cell count 29 × 10^6^/L, protein 733 mg/L). A diagnosis of post-infectious Guillain–Barré syndrome was established. The patient received a 5-day course of intravenous immunoglobulin (IVIG) at 400 mg/kg/day (25 g daily), along with ongoing rehabilitation therapy. .

**Table 1:** Motor nerve conduction study.

Motor	Site	Latency (ms)	Amplitude (Mv)	Distance (mm)	Lat diff (ms)	Velocity (m/s)
Ulnar nerve	Wrist	2.46	8.3	–	2.46	–
	A.Elbow	5.73	6.8	150	3.27	46
	B.Elbow	7.88	6.8	105	2.15	49
Median nerve	Wrist	4.31	1.3	–	4.31	
	Elbow	7.77	1.1	190	3.46	55
	Axilla	9.81	1.4	115	2.04	56
Tibial nerve	Ankle	6.58	0.2	–	6.58	–
	Fib head	14.71	0.1	385	8.13	47
Peroneal nerve	Ankle	–	–	–	–	–
	Fib head	–	–	–	–	–

**Table 2:** Autoantibody testing for peripheral neuropathy.

Project	Result	Project	Result
Anti Sulfatide IgG antibody	**+**	Anti Sulfatide IgM antibody	–
Anti-GM1 antibody IgG	**+**	Anti-GM1 antibody IgM	–
Anti-GM2 antibody IgG	–	Anti-GM2 antibody IgM	–
Anti-GM3 antibody IgG	–	Anti-GM3 antibody IgM	–
Anti-GM4 antibody IgG	–	Anti-GM4 antibody IgM	–
Anti-GD1a antibody IgG	–	Anti-GD1a antibody IgM	–
Anti-GD1b antibody IgG	–	Anti-GD1b antibody IgM	–
Anti-GD2 antibody IgG	–	Anti-GD2 antibody IgM	–
Anti-GD3 antibody IgG	–	Anti-GD3 antibody IgM	–
Anti-GT1a antibody IgG	**+**	Anti-GT1a antibody IgM	–
Anti-GT1b antibody IgG	–	Anti-GT1b antibody IgM	–
Anti-GQ1b antibody IgG	–	Anti-GQ1b antibody IgM	–

### 2.5 Outcome and follow-up

**Figure 4:**
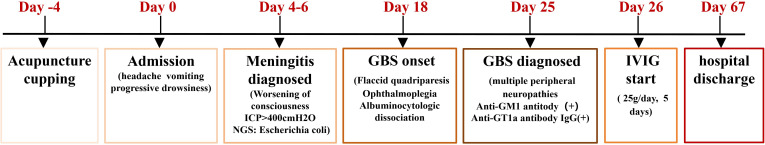
Clinical timeline of the patient's disease course.

The clinical timeline of the patient’s disease course is presented in **Figure 4**. Following IVIG therapy, the patient’s neurological symptoms gradually improved, manifesting as complete eye opening and recovery of muscle strength to grade IV in the upper limbs and grade III in the lower limbs. During planned tracheostomy decannulation, bronchoscopy revealed severe tracheal stenosis located 3 cm proximal to the stoma, leading to the decision to retain the tracheostomy tube. The patient was discharged on hospital day 67 for continued outpatient rehabilitation. One month after discharge, she returned for follow-up to evaluate the feasibility of decannulation. CSF findings are summarized in **Table 3**.

**Table 3:** Changes in cerebrospinal fluid (CSF) parameters.

Admission time (day)	opening pressure (cmH2O)	Appearance	Pandy’s test	Total cell count (x10^6^/L)	White blood cell count (x10^6^/L)	Protein quantification (mg/L)
1	>400	Yellow and turbid	+++	7860	7480	7641
4	>400	Light yellow and slightly turbid	+++	14400	3870	4031
6	370	Light yellow and slightly turbid	+++	2700	1800	2590
9	250	Bloody	++	27000	680	1210
12	230	Pinkish and turbid	++	7830	520	1485
19	345	Light yellow and slightly turbid	+	990	3	777
27	290	Light yellow and clear	+	46	29	733
34	200	Light yellow and clear	+	32	7	718

## 3 Discussion

This case illustrates a complex, sequential interaction between a severe pyogenic infection and a subsequent autoimmune neuropathy in an immunocompromised host. The patient’s course underscores the critical importance of considering new neurological complications even when an initial life-threatening condition appears to be resolving. Our discussion focuses on four key aspects: the postulated pathogenesis, the diagnostic challenge of distinguishing GBS from alternative causes, the unusual nature of the triggering organism, and the clinical implications of this case.

### 3.1 Pathogenesis: from local iatrogenic infection to systemic autoimmunity

This case demonstrates a clear pathway from skin breach to central nervous system (CNS) infection, ultimately triggering an autoimmune neuropathy. Acupuncture and cupping served as the identifiable iatrogenic routes of infection. Although the risk is minimal with standardized procedures, use of unclean instruments or inadequately disinfected skin can lead to inoculation with Escherichia coli. In immunocompetent individuals, such local bacterial invasion is typically cleared effectively. However, this patient—with SLE, chronic renal failure, and long-term corticosteroid use—was immunocompromised, with impaired cellular and humoral immunity, rendering her highly susceptible to opportunistic infections. This clinical profile aligns with previously reported patients with intracranial E. coli infections, who typically had underlying immunodeficiencies, recent surgery or trauma, or contiguous infectious foci (3, 4).

Lumbar imaging in this patient revealed gas collection within the L1 vertebral body and adjacent soft tissues, a finding highly suggestive of infectious spondylitis. This likely served as the initial local suppurative focus following percutaneous bacterial inoculation. Subsequent hematogenous spread or direct extension led to purulent meningitis. Thus, the infection pathway can be summarized as follows: unclean invasive procedure → skin barrier disruption → E. coli inoculation into deep lumbar tissue → suppurative spondylitis → hematogenous or direct dissemination → purulent meningitis, representing a classic iatrogenic infection. The progressive drowsiness on day 4 was attributed to raised intracranial pressure secondary to severe pyogenic meningitis, as evidenced by CSF opening pressure >400 mmH_2_O and imaging findings of cerebral edema. GBS, which developed later, did not contribute to the alteration of consciousness. Similar complications following acupuncture and cupping, including cervical spinal epidural abscess and infectious discitis, have been documented in previous case reports, underscoring that although these traditional practices are generally safe, they carry infection risks that are amplified in immunocompromised hosts (5). A recent report of methicillin-resistant Staphylococcus aureus-induced discitis following acupuncture further emphasizes that bacterial inoculation via acupuncture can lead to deep spinal infections with severe neurological sequelae (6).

The subsequent development of GBS exemplifies post-infectious molecular mimicry. Structural components of Gram-negative bacteria, such as lipopolysaccharides (LPS), share antigenic epitopes with gangliosides on peripheral nerve axons and Schwann cells (7, 8). This similarity can trigger a cross-reactive immune response, wherein pathogen-directed antibodies mistakenly attack peripheral nervous tissue, leading to demyelination and/or axonal injury. The detection of anti-GM1 and anti-GT1a IgG antibodies in our patient provides direct serological support for this mechanism. Anti-GM1 antibodies are frequently associated with acute motor axonal neuropathy and preceding Campylobacter jejuni infection (9). Anti-GT1a antibodies are often implicated in the pharyngeal-cervical-brachial variant of GBS (10). Although molecular mimicry is best established in C. jejuni-associated GBS (11). Emerging evidence suggests it may extend to other Gram-negative pathogens. Regarding the specific E. coli strain in this case, CSF next-generation sequencing identified E. coli-specific sequences (815 reads) but did not perform serotyping or whole-genome sequencing; therefore, the precise strain (e.g., EPEC, STEC) could not be determined—a limitation of the clinical NGS assay. Nevertheless, previous reports have linked certain E. coli strains to GBS via mimicry: EPEC gastroenteritis has induced a GBS variant with elevated anti-GQ1b/anti-GM1 antibodies (similar to our patient) (12), and an ESBL-producing E. coli urinary tract infection has triggered GBS responsive to IVIG (13). Moreover, glycoengineering studies have demonstrated that the GM3 ganglioside epitope can be expressed on the E. coli surface, providing direct experimental evidence for its molecular mimicry capacity (14). Thus, while direct evidence for E. coli-mediated mimicry is limited, structural similarities among Gram-negative LPS/lipooligosaccharides offer a plausible biological basis. The patient’s immunocompromised status (SLE with B-cell hyperreactivity and impaired regulatory T-cell function (15), plus chronic corticosteroid use) may have lowered the threshold for breaking self-tolerance, enabling cross-reactive anti-ganglioside antibodies that might otherwise remain subclinical.

### 3.2 Diagnostic delineation: GBS vs. SLE flare and critical illness neuropathy/myopathy

The diagnostic pivot toward GBS was crucial. Although SLE is associated with various neuropathies, the acute onset of flaccid, areflexic quadriparesis with ptosis was atypical for primary SLE manifestation. SLE-related neuropathy is often more insidious and sensorimotor in nature, and rarely exhibits the classic albuminocytologic dissociation seen in GBS (16, 17). Furthermore, the electrophysiological findings of acute demyelinating polyneuropathy are hallmark features of GBS and distinct from most SLE-related neuropathies (18). The patient’s positive response to IVIG, a standard therapy for GBS, further supports this diagnosis over an SLE flare, which would typically require more aggressive immunosuppression (19).

The distinction between GBS and SLE-associated neuropathy carries profound therapeutic implications. In a patient with SLE who presents with acute paralysis, the initial inclination might be to escalate immunosuppression with high-dose corticosteroids, a mainstay of SLE flare management (20). However, accumulating evidence indicates that glucocorticoids are not beneficial as monotherapy for GBS and do not alter disability outcomes. A systematic review and meta-analysis of six trials involving 587 participants found no significant difference in disability grade between glucocorticoid-treated GBS patients and those not receiving glucocorticoids (21). Instead, plasma exchange and intravenous immunoglobulin (IVIG) are the evidence-based first-line therapies for GBS, with randomized controlled trials demonstrating that both modalities hasten recovery (22). The patient’s positive response to IVIG in this case therefore supports the diagnosis of GBS rather than an SLE flare, which would not be expected to respond to IVIG alone and would typically require more aggressive immunosuppression such as cyclophosphamide or mycophenolate mofetil. Conversely, in SLE-associated fulminant neuropathy that mimics GBS, treatment with standard GBS therapies may yield minimal to no response, necessitating chronic immunosuppressive regimens including high-dose corticosteroids, cyclophosphamide, or rituximab. This therapeutic dichotomy underscores the critical importance of accurate diagnostic differentiation.

Another important differential diagnosis was critical illness polyneuropathy (CIP) or myopathy (CIM), given the patient’s prolonged ICU stay and sepsis. However, CIP/CIM typically presents as difficulty weaning from mechanical ventilation rather than new, progressive weakness after initial neurological improvement and does not feature ptosis or marked albuminocytologic dissociation (23). The elevated CSF protein level and specific antibody profile were pivotal in confirming GBS as the primary cause of paralysis (7).

### 3.3 Characteristics and implications of GBS following *E. coli* meningitis

Campylobacter jejuni is the most extensively studied and common bacterial trigger for GBS, accounting for approximately 30% of cases (24). The present case adds to the previously limited literature on GBS associated with Escherichia coli. Previous reports have primarily linked GBS to E. coli urinary tract or gastrointestinal infections. For instance, a 75-year-old woman with prior acute motor axonal neuropathy developed recurrent GBS manifesting as limb weakness and areflexia ten days after the onset of fever and dysuria following an E. coli urinary tract infection (25). Another patient presented with progressive bilateral lower extremity weakness after an extended-spectrum beta-lactamase-producing E. coli urinary tract infection, which improved significantly after five days of IVIG treatment (2). However, GBS directly triggered by E. coli meningitis is extremely rare. To our knowledge, only a limited number of cases of GBS following bacterial meningitis have been documented in the literature, and most of these involved other pathogens (e.g., Neisseria meningitidis) or complications of suppurative otitis media (26).

Based on this case and the limited literature, such GBS may exhibit the following characteristics. First, timing of onset: It may occur during treatment or recovery from the primary infection, differing from the typical 1–3 week latency after C. jejuni infection, suggesting that sustained antigenic exposure from severe intracranial infection may accelerate the immunopathological process. Second, antibody profile: As seen in this case, multiple antibodies such as anti-GM1 and anti-GT1a may co-occur, potentially associated with a more complex clinical phenotype (e.g., with ophthalmoparesis). Third, diagnostic challenge: Against the backdrop of severe meningitis, flaccid paralysis from GBS can easily be misattributed to unresolved CNS injury or CIP, leading to diagnostic delay. Finally, complex underlying conditions: GBS often occurs in patients with underlying diseases or immunocompromised states, complicating differential diagnosis and clinical assessment.

Notably, this case also featured a lumbar infectious focus (spondylitis). This local lesion not only served as the portal for bacterial invasion into the CNS but also, through persistent inflammation, might have directly stimulated or affected adjacent nerve roots. Theoretically, this could alter the local immune microenvironment, providing a “soil” for the autoimmune response against peripheral nerves. Although speculative, this association suggests that in evaluating similar cases, the relationship between local infection and neuroanatomy warrants attention.

### 3.4 Clinical implications and conclusion

This case offers several critical warnings for clinical practice: First, for practitioners and the public: Invasive traditional therapies such as acupuncture and cupping must be performed by properly trained professionals using strict aseptic technique. For immunocompromised individuals (e.g., those with autoimmune diseases, on immunosuppressants, elderly, or diabetic), the increased infection risk should be fully disclosed, and extra caution should be exercised regarding hygienic environment and sterile equipment. Second, for clinicians: (a) Be vigilant for iatrogenic infections: Consider iatrogenic infection in patients presenting with neurological symptoms following invasive procedures, especially in immunocompromised hosts. (b) Monitor for neurological complications dynamically: Resolution of a severe systemic infection like meningitis does not preclude secondary neurological catastrophes. If a patient develops new, progressive, flaccid paralysis inconsistent with initial CNS infection signs, GBS must be high on the differential diagnosis, regardless of whether the pathogen is a classic antecedent. Early neurological consultation, electrophysiological studies, and CSF analysis for albuminocytologic dissociation are essential. (c) Clarify diagnosis in complex contexts: The presence of an underlying autoimmune disease should not prematurely exclude a concurrent, distinct autoimmune neuropathy like GBS, which requires its own specific immunomodulatory therapy (e.g., IVIG).

In conclusion, we report a rare case of GBS following E. coli meningitis. This case emphasizes that the resolution of meningitis does not preclude the development of new, severe neurological complications. In patients presenting with acute flaccid paralysis during or after treatment for a systemic infection, GBS should be considered even if the triggering microorganism is not a classic antecedent. Early recognition and timely specific immunotherapy with IVIG or plasma exchange are crucial for improving outcomes.

## Data Availability

The raw data supporting the conclusions of this article will be made available by the authors, without undue reservation.
